# A scoring system for the evaluation of the mutated *Crb1/rd8*-derived retinal lesions in C57BL/6N mice

**DOI:** 10.12688/f1000research.11252.1

**Published:** 2017-03-31

**Authors:** Danilo Concas, Heather Cater, Sara Wells

**Affiliations:** 1 Mary Lyon Centre, Harwell Campus, MRC Harwell Institute, Oxfordshire, OX11 0RD, UK

**Keywords:** mouse phenotyping, Crb1/rd8 mutation, retina degeneration

## Abstract

As part of the International Mouse Phenotyping Consortium (IMPC) programme, the MRC Harwell is conducting a large eye morphology phenotyping screen on genetically modified mice compared to the baseline phenotype observed in the background strain of C57BL/6NTac. The C57BL/6NTac strain is known to carry a spontaneous mutation in the *Crb1* gene that causes retinal degeneration characterized by the presence of white spots (flecks) in the fundus. These flecks potentially represent a confounding factor, masking similar retinal phenotype abnormalities that may be detected in mutants. Therefore we investigated the frequency, position and extent of the flecks in a large population of C57BL/6NTac mice to provide the basis for evaluating the presence of flecks in mutant mice with the same genetic background. We found that in our facility males were more severely affected than females and that in both males and females the most common localisation of the flecks was in the inferior hemicycle of the fundus.

## Introduction

**Figure 1.**
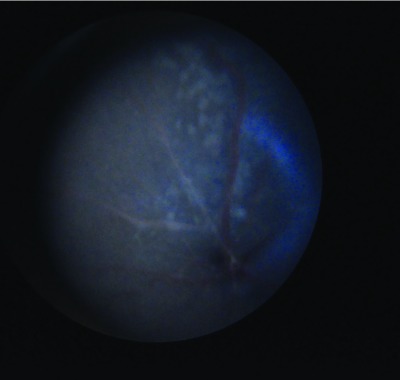
Picture showing the retinal fundus with an example of the flecks treated in this study. The flecks appear in the superior hemicycle of the fundus because the image is inverted by the ophthalmoscope.

Retinal degeneration in mice occurs in many forms, many of which can be attributed to mutations in specific genes. Some of the reported types of retinal degeneration display a similar phenotype, characterised by the presence in the fundus of white spots of different shapes and sizes ^1– 3^. One of the causative mutations for retinal degeneration in the mouse is the spontaneous single nucleotide deletion rd8 in the *Crb1* gene, situated on chromosome 1 ^1, 4^. It has been previously reported that the C57BL/6N strain, derived from the unaffected C57BL/6J strain, often presents typical retinal white spots (flecks) caused by the *Crb1/rd8* mutation ^4^. These have been described as dysplastic lesions affecting the retinal region between the inner and the outer nuclear layer and are mainly localised in the inferior part of the retina ^5, 6^. The observed phenotype is considered a possible confounding factor that could mask a phenotype with a similar appearance but a different causative gene mutation ( Figure 1). This is of particular importance considering that the C57BL/6N line is a widely used commercial line and is the background strain used for the generation of gene-targeted mice in several mouse mutagenesis/phenotyping programmes, including the International Knockout Mouse Consortium ( IKMC) and the International Mouse Phenotyping Consortium ( IMPC).

Over the last 5 years of phenotyping mice through the IMPC pipeline at MRC Harwell, we have observed the presence of fundus flecks in both the knockout lines and in the C57BL/6NTac wild type mice. The number of affected individuals for each knockout line generated has been variable, as has the number of flecks present in each individual. As a result of such variability, the probability that the flecking in the mutant line is a phenotype attributable to the gene mutations rather than the background strain effect becomes questionable. To correctly interpret similar phenotypes in the knockout lines and exclude the contribution of *Crb1/rd8* -related flecks, we have created a scoring system to allow us to fully categorise the lesions present in the C57BL/6NTac mice in a systematic manner in order to provide a comprehensive background strain reference. The flecks scoring system takes into account the position of the flecks in the superior and inferior retinal hemicycle, as the retina is not uniformly affected by the phenotype ^5^. As an innovative approach, we also scored the number of flecks in each hemicycle as a measure of the phenotypic penetrance. In addition, in order to determine any sexual dimorphism, we applied our scoring system to both males and females.

## Methods

### Animals

194 C57BL/6NTac males and 200 females were screened at 15 weeks of age. Animals were housed in IVC cages from birth under 12-hour-on/12-hour-off cyclic lighting, at controlled temperature (21 ± 2°C) and humidity (55 ± 10%) conditions. The mice had free access to water (25 p.p.m. chlorine) and were fed *ad libitum* on a commercial diet (SDS Rat and Mouse No.3 Breeding diet RM3). All procedures and animal studies were carried out in accordance with the Animals (Scientific Procedures) Act 1986, UK, SI 4 2012/3039) and with the NC3R’s ARRIVE guidelines All animal work reported in this article has been optimised to minimise the animals’ suffering and unnecessary procedures.

### Fundus imaging and flecks scoring

For the fundus examination an Omega 180 ophthalmoscope (Heine Ltd, USA) and a Superfield NC lens (Volk Optical Inc., USA) were used. Each eye pupil was dilated using a drop of 1% w/v Minims Tropicamide (Bausch & Lomb Inc., USA) and the observation was performed after 2 minutes. Images of the fundus were acquired by the use of a topical endoscopy fundus imaging (TEFI) camera.

**Figure 2.**
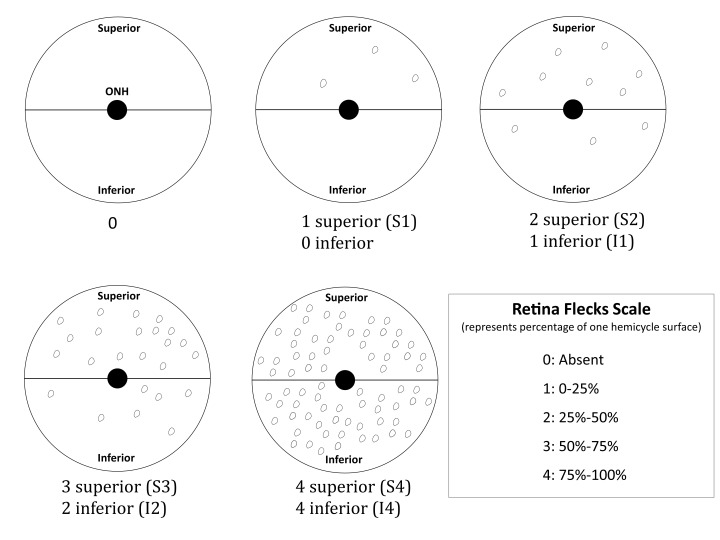
In-house flecks scoring system. The retinal fundus has been divided into two hemicycles: inferior and superior. In each one of the hemicycles the percentage of the surface that is covered by flecks represents the severity grade within a range of 25% for each level. The combination of the position, superior (S) or inferior (I), and the severity grade (from 0 to 4) represents the flecks score.

The examinations were conducted by trained technicians on both eyes and the flecks on individual eyes were evaluated according to an in-house scoring system ( Figure 2), taking into account their position in the fundus with respect to the optic nerve head (superior or inferior) and their number (with respect to the retinal surface covered by the flecks) as a measure of the severity grade. Therefore, the combination of both the position and the severity grade formed a scoring category for each eye.

### Data analysis

All observational data were recorded on a Microsoft Office Excel spreadsheet, and counts and percentage calculations were performed. Where different flecking scores were obtained for the left and right eye of the same animal, the eye with the most severe grade was used for the percentage calculations.

## Results

**Figure 3.**
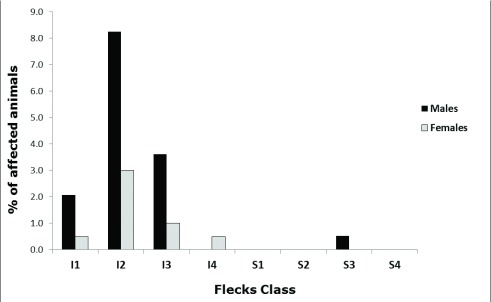
Frequency distribution of the flecks in male and female C57BL/6NTac mice according to our score system. The chart shows the percentage distribution of the flecks in the retinal fundus of males (black columns) and females (white columns) C57BL/6NTac mice. The horizontal categories represent the flecks class as previously explained in Figure 2.

As shown in Table 1, the total percentage of affected males was higher than that of affected females (14.4% of males and 5% of females). Further categorising the flecks according to our scoring system, we observed that the males were still the most affected in the score classes ranging from I1 to I3 ( Figure 3), with a symmetrical distribution of the frequencies centred in the I2 class (25–50% of inferior retina surface) in both sexes (8.2% of males and 3.0% of females). In the sample, there were no males affected in the class I4 (75 to 100% of inferior retina surface), whilst only one female (0.5% of the total) presented that severity grade. We mentioned above that the presence of this kind of flecks has already been associated with the inferior hemicycle of the fundus by other authors, a fact supported by our data that show just one male in the S3 (50–75% of superior retina surface) class of flecking.

**Table 1.** Number of individuals within each flecks class in male and female C57BL/6NTac mice. For each flecks class, the percentage relative to the total number of animals in each sex group has been calculated.

Flecks class	Males affected	Females affected	% of males affected	% of females affected
I1	4	1	2.1	0.5
I2	16	6	8.2	3.0
I3	7	2	3.6	1.0
I4	0	1	0.0	0.5
S1	0	0	0.0	0.0
S2	0	0	0.0	0.0
S3	1	0	0.5	0.0
S4	0	0	0.0	0.0
Total	28	10	14.4	5.0

Flecks scores raw data Click here for additional data file. Copyright: © 2017 Concas D et al. 2017 Data associated with the article are available under the terms of the Creative Commons Zero "No rights reserved" data waiver (CC0 1.0 Public domain dedication).

## Conclusions

With this study we make available both the observational data on the retinal flecks in C57BL/6NTac mice determined by the use of our scoring system, and the scoring system itself. Our findings, using a large population of wild type mice, provide a reference baseline that could significantly contribute to the further evaluation of *Crb1* mutations-based eye morphology phenotypes. In addition to the flecks distribution data, the scoring system used represents a reliable quantitative method to evaluate the degree of flecking of an affected mouse retina and to make the comparison process between two or more strains (or treatment groups) more accurate and manageable.

## Data availability

The data referenced by this article are under copyright with the following copyright statement: Copyright: © 2017 Concas D et al.

Data associated with the article are available under the terms of the Creative Commons Zero "No rights reserved" data waiver (CC0 1.0 Public domain dedication). 

**Dataset 1: Flecks scores raw data.** A spreadsheet with the raw data related to the manual scoring of flecks made by the trained technicians, according to our in-house scoring system. The spreadsheet contains one column related to the animal ID and one column with the score class for males and females. The score class represents the combination of the position, superior (S) or inferior (I), and the severity grade (from 0 to 4) as described in Figure 2.

DOI, 10.5256/f1000research.11252.d156405 ^7^
