# Supplementary material for: A scoring system for the evaluation of the mutated Crb1/rd8-derived retinal lesions in C57BL/6N mice
Source: F1000Res. 2017 Mar 31;6:404. [Version 1] doi: 10.12688/f1000research.11252.1 (PMC5580417; doi:10.12688/f1000research.11252.1)
Supplement: Peer review history

# Peer review: Referee response for version 1

**DOI:** 10.5256/f1000research.12139.r24563

**Published:** 2017-08-29

**Authors:** Michel J. Roux

**Affiliations:** 1 Translational Medicine and Neurogenetics, Institute of Genetics and Molecular and Cellular Biology (IGBMC), CNRS UMR 7104 (French National Centre for Scientific Research), INSERM U 964 (French Institute of Health and Medical Research), University of Strasbourg, Strasbourg, France

The present report describes the distribution of rd8 lesions found in commercial C57Bl/6NTac mice. While the presence of such lesions has been known for a while (Mattapallil et al. 2012, the uncited Simon et al. Genome Biology 2013 ^1^), it is to my knowledge the first such quantitative description, with as well the first report of the higher prevalence of lesions in males compared to females.

 I would suggest to replace Figure 1 with a composite fundus image indicating the extent of the retinal field which is examined (an approximate angle should be indicated), as the retina is rarely observed up to the ciliary margin, especially in a first-line phenotyping as the IMPC pipeline. As some animals may have only peripheral lesions, they may or may not be taken into account depending on the retinal field examined.

 As animals are essentially affected in the inferior hemicycle, I think Figure 2 is misleading. The panels should rather be 0, I1/S0, I2/S1, I3/S2 and I4/S4. It would also be better to include fundus images that illustrate the various grades of scoring.

 The difference between males and females is large, and considering the number of animals, should be significant, but it would be better to use statistics.

 The flecks scores raw data are provided, but it would be useful to provide a comprehensive set of images illustrating the full spectrum of lesions.

 Were the lesions randomly distributed in cohorts, or were some cohorts more affected than others?

I have read this submission. I believe that I have an appropriate level of expertise to confirm that it is of an acceptable scientific standard.

## References

[1] SimonMMGreenawaySWhiteJKFuchsHGailus-DurnerVWellsSSorgTWongKBeduECartwrightEJDacquinRDjebaliSEstabelJGrawJInghamNJJacksonIJLengelingAMandilloSMarvelJMezianeHPreitnerFPukORouxMAdamsDJAtkinsSAyadiABeckerLBlakeABrookerDCaterHChampyMFCombeRDanecekPdi FenzaAGatesHGerdinAKGoliniEHancockJMHansWHölterSMHoughTJurdicPKeaneTMMorganHMüllerWNeffFNicholsonGPascheBRobersonLARozmanJSandersonMSantosLSelloumMShannonCSouthwellATocchini-ValentiniGPVancollieVEWesterbergHWurstWZiMYalcinBRamirez-SolisRSteelKPMallonAMde AngelisMHHeraultYBrownSD: A comparative phenotypic and genomic analysis of C57BL/6J and C57BL/6N mouse strains. *Genome Biol*.2013;14(7) : 10.1186/gb-2013-14-7-r82 R82 10.1186/gb-2013-14-7-r82 23902802PMC4053787

# Peer review: Referee response for version 1

**DOI:** 10.5256/f1000research.12139.r23403

**Published:** 2017-06-12

**Authors:** Cheryl Mae Craft

**Affiliations:** 1 USC Roski Eye Institute, Arcadia, CA, USA; 2 Department of Ophthalmology, Keck School of Medicine, University of Southern California, Los Angeles, CA, USA

The report is interesting in describing the Crb1 /Rd 8 phenotype; however, the genetic analysis is still essential to use and to verify the status of the mutation since other genetic defects can lead to a similar retinal phenotype on different mouse background strains.

 Limited references were included. 

 Because Crb1/Rd8 is a recessively inherited gene, it can significantly play a role and the defective Crb1 protein can interact with other retinal proteins that lead to degeneration and go undetected because of its regional effect.

I have read this submission. I believe that I have an appropriate level of expertise to confirm that it is of an acceptable scientific standard.
